# M-protein and other intrinsic virulence factors of *Streptococcus pyogenes *are encoded on an ancient pathogenicity island

**DOI:** 10.1186/1471-2164-10-198

**Published:** 2009-04-27

**Authors:** Alexandre Panchaud, Lionel Guy, François Collyn, Marisa Haenni, Masanobu Nakata, Andreas Podbielski, Philippe Moreillon, Claude-Alain H Roten

**Affiliations:** 1 Department of Fundamental Microbiology, University of Lausanne, Quartier UNIL-Sorge, Bâtiment Biophore, CH-1015 Lausanne, Switzerland; 2 Department of Medicinal Chemistry, University of Washington, Seattle, WA, USA; 3 Molecular Evolution, Uppsala University, Norbyvägen 18C, 75236 Uppsala, Sweden; 4 Institute of Microbiology, University Hospital Center and University of Lausanne, Bugnon 48, CH-1011 Lausanne, Switzerland; 5 Agence Française de Sécurité Sanitaire des Aliments (Afssa), Avenue Tony Garnier 31, 69007 Lyon, France; 6 Deptartment of Medical Microbiology, Virology & Hygiene, Univ. Hospital, Schillingallee 70, D-18057 Rostock, Germany; 7 Dept. of Oral and Molecular Microbiology, Osaka University Graduate School of Dentistry, 1 – 8, Yamadoaka, Suita-Osaka 565-0871, Japan

## Abstract

**Background:**

The increasing number of completely sequenced bacterial genomes allows comparing their architecture and genetic makeup. Such new information highlights the crucial role of lateral genetic exchanges in bacterial evolution and speciation.

**Results:**

Here we analyzed the twelve sequenced genomes of *Streptococcus pyogenes *by a naïve approach that examines the preferential nucleotide usage along the chromosome, namely the usage of G versus C (GC-skew) and T versus A (TA-skew). The cumulative GC-skew plot presented an inverted V-shape composed of two symmetrical linear segments, where the minimum and maximum corresponded to the origin and terminus of DNA replication. In contrast, the cumulative TA-skew presented a V-shape, which segments were interrupted by several steep slopes regions (SSRs), indicative of a different nucleotide composition bias. Each *S. pyogenes *genome contained up to nine individual SSRs, encompassing all described strain-specific prophages. In addition, each genome contained a similar unique non-phage SSR, the core of which consisted of 31 highly homologous genes. This core includes the M-protein, other *mga*-related factors and other virulence genes, totaling ten intrinsic virulence genes. In addition to a high content in virulence-related genes and to a peculiar nucleotide bias, this SSR, which is 47 kb-long in a M1GAS strain, harbors direct repeats and a tRNA gene, suggesting a mobile element. Moreover, its complete absence in a M-protein negative group A *Streptococcus *natural isolate demonstrates that it could be spontaneously lost, but *in vitro *deletion experiments indicates that its excision occurred at very low rate. The stability of this SSR, combined to its presence in all sequenced *S. pyogenes *sequenced genome, suggests that it results from an ancient acquisition.

**Conclusion:**

Thus, this non-phagic SSR is compatible with a pathogenicity island, acquired before *S. pyogenes *speciation. Its potential excision might bear relevance for vaccine development, because vaccines targeting M-protein might select for M-protein-negative variants that still carry other virulence determinants.

## Background

Bacteria undergo constant mutations and horizontal gene transfer that help them compete in particular ecological niches. Genetic elements can be transferred on DNA stretches, within viruses, or by intercellular contacts. For example, bacteriophages carrying toxin genes can be inserted into bacterial chromosomes and re-program *Streptococcus pyogenes *to produce streptococcal toxic shock syndrome [1,2], or *Staphylococcus aureus *to express Panton-Valentine toxin [3]. Likewise, plasmids and pathogenicity islands can transform non-pathogenic *Escherichia coli *into virulent enteropathogenic (EPEC) or enterohemorrhagic (EHEC) strains [4-6]. Thus, horizontal gene transfer is critical for bacterial genome evolution, and includes genes for virulence, antibiotic resistance and metabolic features [7-9]. Objective criteria have been established to detect them, especially pathogenicity island: presence of virulence-related genes, location on the chromosome, different G+C content, direct repeats on the flanks, association with a tRNA, presence of mobility genes (integrases, transposases, insertion sequences), ability to be mobilized, site-specific integration [10].

When mobile elements confer an advantage to the recipient, they promote its clonal expansion and may become stabilized in the bacterial host. This is illustrated by the insertion of SCC *mec *(staphylococcal cassette chromosome) into the *S. aureus *chromosome, generating methicillin-resistant *S. aureus *(MRSA) [11-13] which successfully expanded in the hospital and recently in the community [14]. However, when all bacteria of the same type share a similar mobile element, its acquired nature may pass unnoticed. It may ultimately become a taxonomic criterion, thus blurring the history of horizontal gene transfer that shaped important pathogens.

Genome analysis by bioinformatics helps highlight such issues. The approach stands on the fact that the genome architecture differs in distinct living organisms, including at the gene level, the G+C content, the codon usage, and/or more subtle biases in nucleic acid arrangements [15]. Nevertheless, bioinformatic methodologies present limitations. For instance, when a foreign element is shared by most of the strains, the comparison of the gene content of these strains will not identify this element as foreign. Likewise, comparing G+C contents between the core chromosome and a putative mobile element is inconclusive when the recipient's chromosome and the mobile element shared a similar G+C content at the time of the horizontal transfer, or when the G+C content of the mobile element has progressively adapted to that of the recipient, a process called homing [10]. Acquired elements may also be identified by the presence of relics of prophages or DNA mobilization signatures in the core chromosome – e.g. integrases, excisases, or the presence of direct repeats or tRNA genes at the border of the element. However, such signatures might gradually become cryptic by amelioration occurring during island stabilization [16].

The present study uses a naïve approach, called "cumulative TA skew" [17-20] to seek the presence of foreign genetic elements in the genomes of the twelve currently completely sequenced *S. pyogenes *strains (Table 1). This method measures the local nucleotide usage without any a priori on nucleotide composition of DNA sequences. It can differentiate between DNA segments that slightly favor different nucleotide usage. Such approach was developed in cryptography and linguistics [21], enabling for instance to spot an English paragraph within a French text, knowing that English spelling favors the use of the digram "th". Such non a priori approaches, based on letter/nucleotide usage, do not need any information of word/codon, grammar/structure or style/genome to identify pattern differences.

**Table 1:** Completely sequenced strains of *Streptococcus pyogenes *used in this study. M protein type, size, number of SSRs, associated diseases, accession number as well as references are summarized.

**Index**	**Strain**	**M-protein type**	**Size, bp**	**SSRs**	**Accession number**	**Associated disease**	**Reference**
1	SF370	1	1,852,441	5	[GenBank:NC_002737]	Pharyngitis and invasive disease	[26]
2	MGAS5005	1	1,838,554	4	[GenBank:NC_007297]	Sepsis and meningitis	[44]
3	MGAS10270	2	1,928,554	6	[GenBank:NC_008022]		[36]
4	MGAS315	3	1,900,521	7	[GenBank:NC_004070]	Streptococcal toxic shock syndrome	[37]
5	SSI-1	3	1,894,275	7	[GenBank:NC_004606]		[39]
6	MGAS10750	4	1,937,111	6	[GenBank:NC_008024]		[36]
7	Manfredo	5	1,841,271	6	[GenBank:NC_009332]	Acute rheumatic fever	[41]
8	MGAS10394	6	1,899,877	9	[GenBank:NC_006086]		[27]
9	MGAS2096	12	1,860,355	4	[GenBank:NC_008023]	Pharyngitis	[36]
10	MGAS9429	12	1,836,467	4	[GenBank:NC_008021]		[36]
11	MGAS8232	18	1,895,017	6	[GenBank:NC_003485]	Acute rheumatic fever	[40]
12	MGAS6180	28	1,897,573	6	[GenBank:NC_007296]	Puerperal sepsis	[29]

This simple genometric analysis or genome biometrics unambiguously identified all the described *S. pyogenes *prophages, which differed from the core chromosome and were variously distributed in the twelve sequenced chromosomes (Table 1). Moreover, it revealed an additional unique divergence region 47-kb in average (varying from 39 to 53 kb in the different strains), which is conserved in all sequenced strains, and encodes major intrinsic *S. pyogenes *virulence factors, including M-protein and the *mga-*virulon [1,22-24]. It also fairly complies with Hacker's criteria for a pathogenicity island [7-9]. Thus, M-protein belongs to a large pathogenicity island that was probably acquired before the *S. pyogenes *speciation. Its potential instability could have practical implications for species identification in the clinical laboratory. Moreover, since M-protein is a vaccine target, the question arises as to whether anti-M-protein vaccines might select for escape variants lacking M-protein.

## Results

### *S. pyogenes *cumulative nucleotide skews

**Figure 1:**
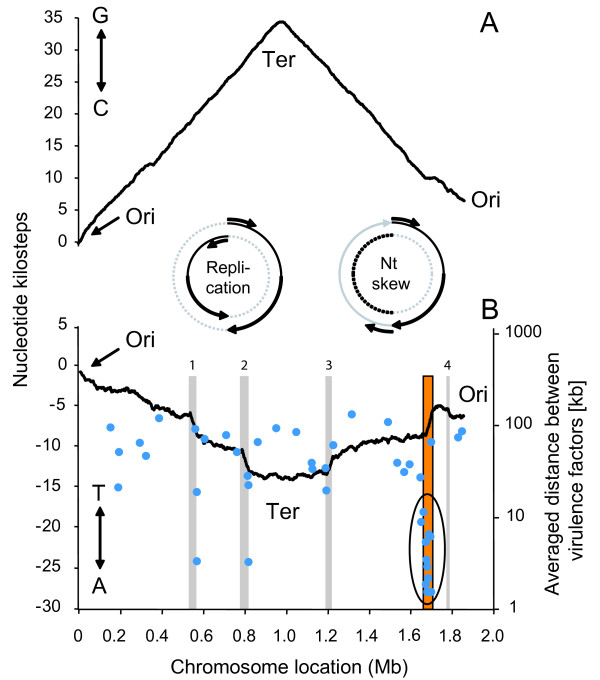
**Cumulative GC skew (A) and TA skew (B) of the chromosome of the M1 *S. pyogenes *SF370**. Cumulative skews (Y-axis, in nucleotide kilosteps) are represented along the chromosome (X-axis). Both skews are performed on one of the two strand of the streptococcal DNA double helix. The TA skew (B) reveals segments with steeper slopes referred to as steep-slope segments (SSRs) and revealing regions with different nucleotide composition. Phagic SSRs are represented by grey rectangles and numbered as they appear on the chromosome, while the conserved non-phagic SSR is outlined by a boxed orange rectangle. Virulence genes (blue dots) are spotted according to their position on the chromosome and the distance to the next virulence gene (right Y-axis, logarithmic scale). The overall average distance between virulence genes is 54.8 kb, except for a group of 10 genes (circled) present in the non-phagic 47-kb SSR for which the average distance is only 4.3 kb. This represents a ten-fold greater concentration of virulence factors compared to the rest of the chromosome.

Fig. 1 presents the cumulative GC- and TA-skew curves of *S. pyogenes *M1 SF370. As represented in the insets, prokaryotes present a bidirectional replication starting from the origin of replication and reading in both directions until reaching a terminus (replication inset). The bidirectional replication therefore defines a leading and a lagging strand in the double helix. In the contrary, nucleotide sequence reading (nt skew inset) is unidirectional, starting from the origin of replication, passing through the terminus and finishing at the origin again (nt skew inset). The cumulative GC-skew (Fig. 1A) is composed of two symmetrical linear segments defined by the origin and the terminus of DNA replication corresponding to the minimum and the maximum of the curve respectively [19,25] (see also Comparative Genometrics [20]). The positive or ascending slope reveals that this part of the chromosome sequence is enriched in guanine residues, whereas the negative or descending slope means that it is enriched in cytosine residues. This symmetric architecture relies on the almost universal bidirectional DNA replication and circular nature of the bacterial chromosome (inset in Fig. 1).

**Figure 2:**
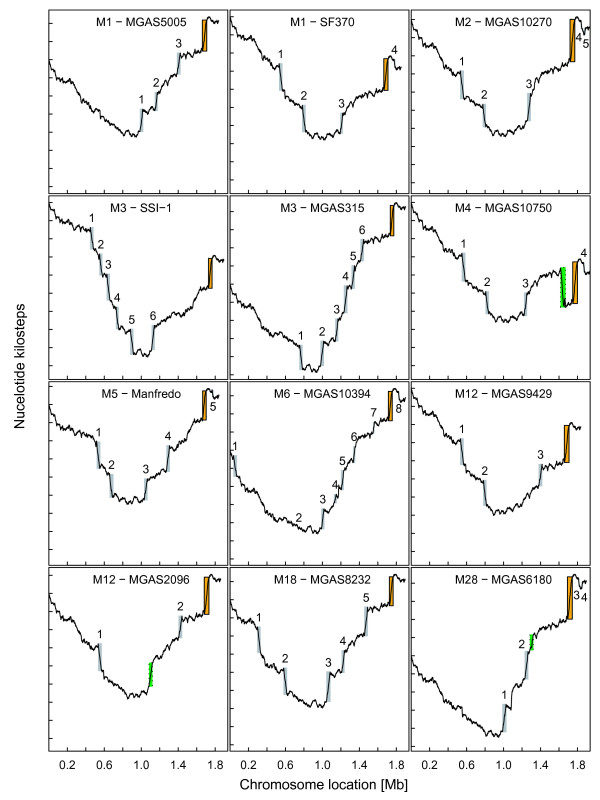
**TA skews (as in Fig. 1) for the twelve available genomes of *S. pyogenes *(see Table 1)**. Each tick mark on the vertical axes indicates 2000 T over A excess. Phagic SSRs are represented by grey rectangles while the conserved non-phagic SSR is outlined by boxed orange rectangles. In MGAS2096, MGAS6180 and MGAS10750, peculiar regions not documented as phages and presenting a different TA skew are shown with a green dotted box. These three regions are discussed in the text. Eventually, SSRs that are not described as prophages, but resemble mobile elements, are also present in the genomes of MGAS10750 and MGAS2096 and are the purpose of further studies. Phage numbering follows the order on the chromosome: in MGAS9429, 1 stands for phage 9429.1, 2 stands for phage 9429.2, etc (see references in Table 1).

The cumulative GC-skews of the other eleven *S. pyogenes *chromosomes are similar to that shown in Fig. 1A (data not presented). In contrast, the cumulative TA-skews are quite different both in terms of smoothness and in inter-strain variability (Fig. 1B and Fig. 2). Although both chromosomal arms conserve their symmetry, their slopes are opposite in TA- and GC-skews. Moreover, the two segments of the TA skew curves are interrupted by segments of steeper slopes, referred to as steep-slopes regions (SSR). These SSRs identify regions exhibiting different nucleotide composition than the core genome, and thus may highlight DNA segments of different origins.

### Genetic content of the SSRs in *S. pyogenes *M1 SF370

The cumulative TA-skew of strain SF370 contains five major SSRs (Fig. 1B). The nucleotide sequence of four of them corresponds to the four prophages (370.1, 370.2, 370.3 and 370.4) described in this strain [26]. The fifth SSR is a 47-kb segment consisting of 40 ORFs (Additional file 1), of which 10 (25%) code for *S. pyogenes *intrinsic virulence factors, including M-protein and part of the *mga *virulon [1,22,23]. The other 30 (75%) code for determinants not known to be involved in pathogenicity, but including features compatible with an ancient mobile elements [10], such as a transposase gene (spy2013), two 11-bp direct repeats (starting at positions 1663812 and 1710243), and the vicinity of Lys-tRNA gene as a putative insertion/excision site. Fig. 1B also presents the distribution of the putative *S. pyogenes *virulence genes along the SF370 chromosome. Out of 43 virulence genes [26], 10 (24%) are concentrated in the 47-kb SSR, 9 (21%) are located within prophages, and 24 (55%) are scattered along the rest of the genome. Thus, the density of virulence genes in the 47-kb SSR (one virulence gene/4.3 kb) is 10-fold higher than in the rest of the chromosome (one virulence gene/54.8 kb), further suggesting a pathogenicity island [7-9].

### Cumulative TA skews of the other sequenced *S. pyogenes *chromosomes

**Figure 3:**
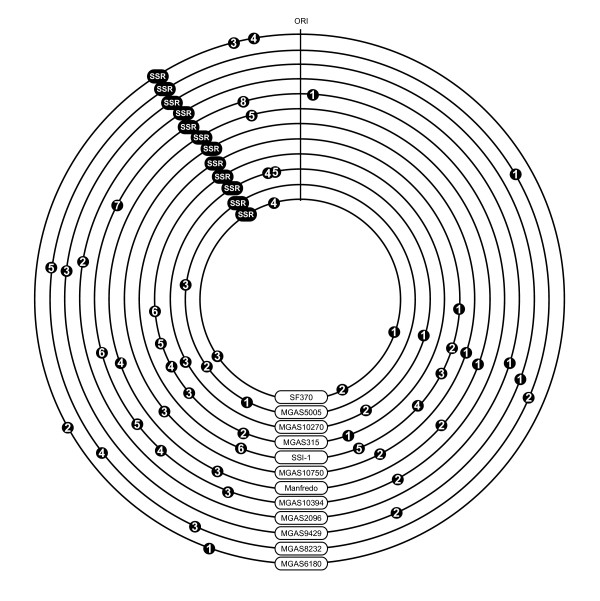
**Location of the SSRs along the twelve *S. pyogenes *chromosome maps**. All phagic and non-phagic SSRs revealed by cumulated TA skews are represented by black symbols. Phages are numbered sequentially according to their position along the chromosome, as already used in Beres *et al*. [37].

Fig. 2 depicts the cumulative TA-skews of the twelve sequenced *S. pyogenes *chromosomes. All cumulative TA-skews display the expected V-shape. In addition, each chromosome presents a unique set of SSRs located at various positions, except for the non-phagic 47-kb SSR whose position is conserved, but whose size varies from 39 to 53 kb. The SSRs located at various places identify the strain-specific prophages already described in *S. pyogenes *(Fig. 2 and Fig. 3). They also spot the composite prophage-transposon chimera carrying an erythromycin-resistance marker and the R6 protein specific to sore-throat *S. pyogenes *M6 strains [27] (phage 4 of MGAS10394 on Fig. 2), as well as the 37.4-kb region of RD2 recently reported in the chromosome of *S. pyogenes *M28, responsible of the puerperal fever [28,29] (see MGAS6180 on Fig. 2). Other SSRs that are not described as prophages, but resemble mobile elements, are also present in the genomes of MGAS10750 (M4) and MGAS2096 (M12) (Fig. 2).

**Figure 4:**
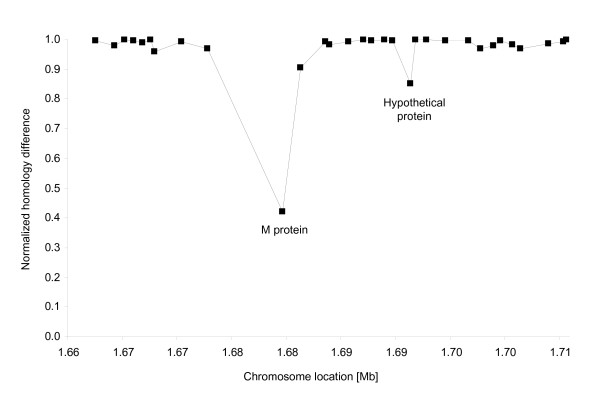
**Similarity comparison of the core 31 proteins present in all the non-phagic 47-kb SSRs of all strains, and missing in the M-protein-negative group A streptococcus T11**. SF370 proteins are compared with proteins of all other genomes present in Table 1. Protein Blast scores for the three genomes were averaged and normalized to the score of M1 versus itself (normalized score of 1). M protein type I (spy2018) showed a major difference in its amino acid composition among strains.

Since the 47-kb SSR is shared by all the strains, we further analyzed the amino acid variations of this element by comparing protein sequences of strain SF370 to those of the eleven other sequenced strains (Table 1). The 47-kb SSR consists of a Lys-tRNA gene and a core of 31 ORFs, plus a few additional genes which vary between the organisms (Additional file 1). The 31 core gene products showed a high similarity (≥ 90% protein similarity), with the notorious exception for the variable M-protein (42%) (Fig. 4) [30,31]. This is expected because the sequenced isolates represent different M-protein serotypes.

**Figure 5:**
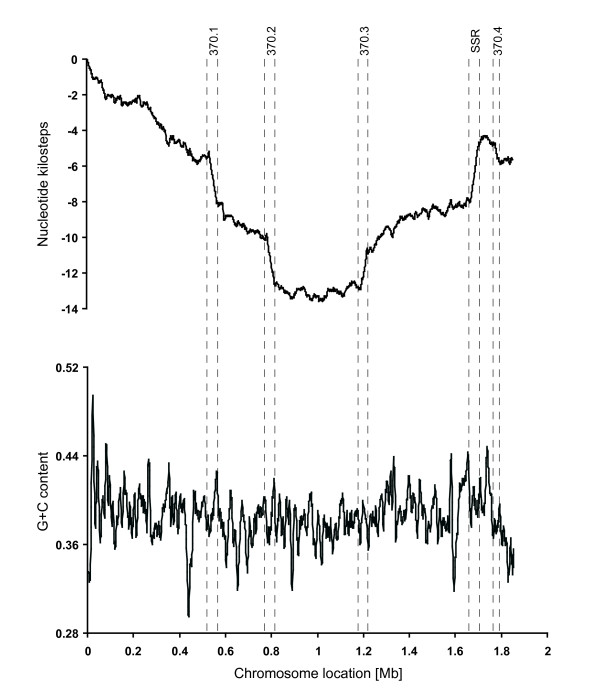
**Comparison of TA skew (A) and G+C content (B) of *S. pyogenes *SF370**. SSR positions in the TA skew as well as in the G+C content are shown using dashed lines.

Thus, the genometric analysis identified all the horizontally-acquired *S. pyogenes *prophages, plus a unique non-phagic SSR compatible with a pathogenicity island. For comparison, the G+C content analysis did not detect any of these elements (Fig. 5).

### Detection of spontaneous loss of the 47-kb SSR

**Figure 6:**
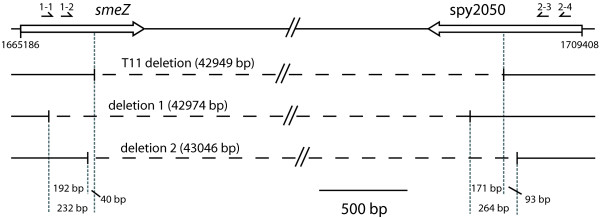
**Spontaneous excision of M1 *S. pyogenes *SF370**. Mitogenic exotoxin Z (*smeZ*/spy1998) and putative PTS system enzyme IIC component (spy2050) are the two coding genes where the boundaries for the natural isolate of M-protein-negative group A streptococcus T11 can be found (T11 deletion). In two out of four individual cycled cultures, an amplicon compatible with the excision of most part of the 47-kb SSR element was detected as shown by the two deletions (deletion 1 and deletion 2). Both amplicons were 200 bp apart from the boundaries of the natural T11 isolates. The region defined by the TA skew analysis starts 4520 bp upstream of the 5'-end of *smeZ*, and ends 835 bp downstream of the 3' end of spy2050 (see additional file 1). Oligonucleotides SVC1-1, SVC1-2, SVC2-3, SVC2-4 are represented above the genes. A 0.5-kb scale is indicated.

Since the M-protein-containing 47-kb SSR might represent a horizontally acquired element, we sought both whether natural group A streptococci missing this segment might exist, and whether it could be lost from *S. pyogenes *grown in vitro. A natural isolate of M-protein-negative group A streptococcus (serotype T11) [32] was analyzed. PCR-amplification indicates that this strain lacks a large 43-kb region, which encompasses 92% of the 47-kb SSR as determined by its genometric boundaries (Additional file 1 and Fig. 6).

Spontaneous deletion was sought by PCR-amplification of the whole 47-kb SSR from genomic DNA prepared from liquid cultures of reference strain SF370. In two out of four individual cycled cultures, an amplicon compatible with the excision of most part of the 47-kb SSR was detected (Fig. 6). DNA sequencing indicate that the loss had occurred between *smeZ *(spy1998) and spy2050, corresponding to the region missing in the M-protein-negative T11 within a variation of 200 bases on each side (Additional file 1 and Fig. 6). Since DNA was extracted from batch culture of ca 10^10^–10^11 ^colony forming units, spontaneous deletion occurred at a frequency estimated higher than 10^-11^.

## Discussion

*S. pyogenes *is a highly versatile pathogen, which produces suppurative infections, toxin-related diseases, and delayed non-suppurative sequels [2,33,34]. A key element in its virulence is M-protein, a coil-coil peptidoglycan-attached polypeptide conferring anti-phagocytic properties. M-protein belongs to an *emm *and *emm*-like gene family, and is characterized by a conserved C-terminal anchored in the cell wall, successively followed by conserved C-repeats, variable B-repeats and hypervariable A-repeats [30,31]. These variable repeats are responsible for > 125 different M-serotypes [35].

Few M-serotypes are preferentially represented in certain disease strains [1]. Recently, serotype M1 was associated with pharyngitis and invasive diseases [26], M12 with pharyngitis [36] M3 with streptococcal toxic shock syndrome [37-39], M6 with pharyngitis and macrolide-resistance due to the *mefA *gene [27], M5 and M18 with acute rheumatic fever [40,41], and M28 with puerperal fever [28,29]. Yet, M-protein alone does not account for the whole spectrum of *S. pyogenes *infections. Up to 40 additional virulence genes are involved, which are encoded either on the streptococcal core chromosome or on prophages or transposons inserted in it [26].

Lately researchers analysed the genomic peculiarities of specific epidemic *S. pyogenes *strains, and compared them to collections of epidemiologically-related and unrelated isolates [26,27,29,37-40]. All strains exhibited a highly conserved core genome constituted of ca. 1.7 Mb, with a 38.4–38.7% G+C content, and a high (≥ 90%) nucleotide similarity. In addition, epidemiologically-related strains presented similar assortments of horizontally-acquired genetic elements, including mostly – but not exclusively – prophages that carried super-antigens, surface adhesins and sometimes antibiotic (macrolides)-resistance genes [27]. One salient example is the region of divergence RD2 recently described in a puerperal fever-related serotype M28 *S. pyogenes *strain [28,29]. RD2 is a large insert that is absent from other *S. pyogenes *serotypes, but was found in *Streptococcus agalactiae*, which also colonizes the female genital tract and can produce neonatal infections. RD2 encodes a transposase as well as surface adhesins that are involved in adherence to genito-urinary mucosal cells [28]. Thus, it is likely to be an acquired element that is responsible for the niche-related puerperal fever produced by the serotype M28 and related strains.

Ferretti et al. [26] showed that serotype M1 strain SF370 carried 43 putative virulence genes, of which 34 (79%) are located on the core genome and 9 (21%) on prophages. Comparative genomics indicated that the virulence genes of the core chromosome are highly conserved in the sequenced strains, and thus are likely to provide *S. pyogenes *with its basal virulence capability. In contrast, acquired virulence genes are variable and are likely to afford disease specificity [42-44]. The present results add supplementary arguments to the critical role of horizontally acquired genes in the evolution of bacterial pathogens. Indeed the major virulence genes considered species-specific of *S. pyogenes*, are located on a non-phagic 47-kb SSR that carries features of a stabilized pathogenicity island [7-9].

Because of its high inter-strain homology, the evolutionary history of the non-phagic 47-kb SSR is not easy to reconstruct. However, a few hallmarks are apparent. First, the fact that it carries species-specific virulence factors – e.g. M-protein – indicates that it was acquired before the *S. pyogenes *speciation. Second, since it shares the same chromosomal location in all the sequenced strains, it was probably present in the genome before the acquisition of most prophages and other mobile elements, which vary in different strains. Third, since it is highly conserved among all sequenced strains, except for the anti-phagocytic M-protein, it was probably acquired only at a very few occasions, and further evolved different M-protein serotypes due to the immunologic pressure of the host. Eventually, the fact that it carries an identical set of 31 ORFs in all the strains, plus some additional genes in few isolates, suggest that it has further evolve by gene acquisition in these particular strains.

The current relatively large 47-kb SSR is probably difficult to mobilize. This is supported by the fact that the loss of the element occurs neither between direct repeats nor at the Lys-tRNA locus, although the Lys-tRNA gene might have been the primordial insertion site in the chromosome. In pathogenicity islands conferring selective advantages to their host, all elements promoting island excision are progressively lost, leading to their stabilization in the bacterial chromosome [10]. An additional selective advantage conferred by the 47-kb SSR might be the presence of several or all components of a hexose and a dipeptide importer, respectively. Indeed, the dipeptide permease was shown to contribute to bacterial growth and to expression of crucial virulence factors [31].

The high inter-strain conservation and the stability of the 47-kb SSR reflect its ancient acquisition. Nevertheless, accidental loss, probably by RecA-mediated recombination, is possible as supported experimentally, and might be favored by the presence of the direct repeats flanking the 47-kb SSR. The existence of such M-protein-negative strains might be underestimated, since routine identification of *S. pyogenes *determines only the presence of group A polysaccharide, ignoring the presence of M-protein [45]. Thus, it raises several important issues. First for taxonomy, because it is assumed that all group A polysaccharide streptococci carry the M-protein. Second for pathogenesis, because it would be relevant to know the *S. pyogenes *ancestor and how it acquired the M-protein gene. Finally for vaccine development, because a strategy targeting the products encoded by the 47-kb SSR, e.g. M-protein, might select strains having lost the whole region, thus generating M-protein-negative strains that still carry prophage-encoded toxins and adhesin genes.

## Conclusion

Using the cumulative TA skew – a naïve method measuring biases in nucleotide composition – for the first time in this purpose, we could point to all known prophages of the twelve *S. pyogenes *sequenced chromosomes. Moreover, we showed that a region with similar biases, but not identified as a phage, is shared by all the strains, and concentrates one quarter of the known pathogenicity genes in about 50 kb. Missing in at least one natural isolate and experimentally excisable at a very low frequency, this putative ancient pathogenicity island may have been acquired before *S. pyogenes *speciation, and subsequently become stabilized. Taken together, these results may allow to discover new genes involved in pathogenicity, and reinforce the importance of mobile regions on the evolution of pathogenicity in bacteria.

## Methods

### Nucleotide sequences and genometric analyses

Full genome sequences and annotation files of 12 currently sequenced *S. pyogenes *strains (Table 1) were retrieved from the NBCI database [26,27,29,36,37,39-41,44].

We used the algorithms described in [17,19] and implemented in the Genometrician's Scooter and in Comparative Genometrics [20] to investigate along raw chromosome sequences local biases of Gs and Cs, or Ts and As. First GC- and TA-skew values measuring the G and T excesses are determined for each 1-kb window. Next, cumulative GC- or TA-values are calculated for a window *i *by summing to its skew value *Sk*_*i *_all preceding ones from *Sk*_1 _to *Sk*_*i*-1_. Finally, a cumulative curve is drawn by plotting to each position of window center the cumulative skew value [19].

### Bacteria and growth conditions

Bacterial strains included the sequenced M1 *S. pyogenes *SF370 (ATCC 700294) [26], and a natural group A streptococcal isolate presenting a M-protein-negative serotype T11 [32]. Bacteria were identified at the species level by standard diagnosis methods including ribotyping and A-carbohydrate and M-protein typing [1,45]. They were grown without aeration in brain heart infusion broth (BHI; Oxoid Ltd, Hampshire, England) at 37°C, under a 5% CO_2 _atmosphere. Bacterial stocks were kept -80°C in 10% (vol/vol) of glycerol.

### Detecting the loss of a putative 47-kb pathogenicity island

The genometric analysis identified a putative 47-kb pathogenicity island encompassing the M-protein and other virulence genes (see Results section). We tested its possible loss in the M-protein-negative strain T11 and in strain SF370. Genomic DNA was extracted with the Qiagen DNeasy Tissue kit (Qiagen GmbH, Hilden, Germany). To detect a possible excision in strain T11 and determine its precise limits, we amplified DNA over the boundaries of the 47-kb segment by using converging primers synthesized by Microsynth (Balgach, Switzerland) targeting internal and flanking regions of the putative pathogenicity island. In addition to a series of control primer pairs directed to spy1999, spy2000 (open reading frame designation according to strain SF370) on one island side, and spy2039, spy2040, spy2043, spy2045, spy2047, and spy2049 on the other side, two oligonucleotides named SVC1-1 (5'-ACCAATCCGTTGTCCAAA) and SVC1-2 (5'-GGGTAATCCGGGCTATTCAG) were designed as forward primers hybridizing the *smeZ *gene (spy1998 in strain SF370), two others called SVC2-3 (5'-CAGGTGGTGGCACCTTTATT) and SVC2-4 (5'-GTTCCAGCAGAAGGTGAAGC) were selected to target spy2050 as backward primers. Utilizing the different primer combinations on the T11 genomic DNA, PCR cycling conditions consisted of 30 cycles at 94°C for 30 sec, 52°C for 45 sec, and 72°C for 2.5 min, followed by a 10-min delay period at 72°C after the last cycle. Detectable PCR-amplified fragments were first purified with the PCR DNA and gel band purification kit GFX (Amersham Biosciences, Buckinghamshire, England), and then sequenced by Synergen Biotech (Schlieren, Switzerland).

To detect spontaneous excisions of the 47-kb element in strain SF370, the bacterium was grown overnight to stationary phase in four independent cultures cycled ten times in rich medium, from which DNA was prepared and processed for nested PCR according to Lesic *et al *[46] in order to detect very low amounts of DNA. Briefly, a first PCR was performed using primers SVC1-1 and SVC2-4, localized respectively 759 bp upstream and 531 bp downstream of the excision site predicted from protein M-negative strain T11 (see Results section). A second round of amplification was performed using the initial PCR mixture as template and primers internal to the first amplified sequence (SVC1-2 644 bp upstream and SVC2-3 281 bp downstream of the predicted excision site). Final amplification products were purified by agarose electrophoresis and sequenced as above.

## Abbreviations

SSR: steep slope region; MRSA: methicillin-resistant *Staphylococcus aureus*.

## Authors' contributions

CAHR designed the study. APa made the bioinformatic analysis, with a contribution by LG. FC, MH and MN performed the in vitro experiments, designed by PM and APo. APa, LG, PM, and CAHR drafted the manuscript. All authors took part in the interpretation of the results, improved the manuscript and approved its final version.

## Supplementary Material

Additional file 1 **Gene content of the non-phagic 47-kb SSR of M1 *S. pyogenes *SF370.** Click here for file
