# Supplementary material for: M-protein and other intrinsic virulence factors of Streptococcus pyogenes are encoded on an ancient pathogenicity island
Source: BMC Genomics. 2009 Apr 27;10:198. doi: 10.1186/1471-2164-10-198 (PMC2683870; doi:10.1186/1471-2164-10-198)
Supplement: Additional file 1 — Gene content of the non-phagic 47-kb SSR of M1 S. pyogenes SF370. [file 1471-2164-10-198-S1.pdf]

Additional file 1: Gene content of the non-phagic 47-kb SSR of M1 *S. pyogenes* SF370.

*Index**	*5'*	*3'*	*Strand*	*Length [aa]*	*PID*	*Synonym*	*Gene*	*Sharing*	*Product*
	1663725	1663797	+	-	15674250	spyt56	-	All	Lys-tRNA
1	1663936	1664376	+	146	15675783	spy1994	-	2,3,6,7,8,9,10,11,12	pai1 protein (theoretical repressor)
2	1664431	1664937	+	168	15675784	spy1995	*flaR*	2,3,6,7,8,9,10,11,12	topology modulation protein
3	1665186	1665887	+	233	15675785	spy1998	*smeZ*	2,4,6,7,8,9,10,11,12	**mitogenic exotoxin Z**
4	1666171	1666437	-	88	15675786	spy1999	-	2,3,4,5,6,9,10,11,12	hypothetical protein
5	1666616	1668244	+	542	15675787	spy2000	*dppA*	All	surface lipoprotein
6	1668357	1669334	+	325	15675788	spy2001	*dppB*	All	transmembrane transport protein
7	1669331	1670152	+	273	15675789	spy2002	*dppC*	All	transmembrane transport protein
8	1670164	1670967	+	267	15675790	spy2003	*dppD*	All	ATPase protein
9	1670951	1671577	+	208	15675791	spy2004	*dppE*	All	ATPase protein
10	1671658	1671858	-	66	15675792	spy2005	-	All	hypothetical protein
11	1672027	1674504	-	825	15675793	spy2006	-	All	hypothetical protein
12	1674517	1675437	-	306	15675794	spy2007	*lmb*	All	**putative laminin adhesion**
13	1675673	1676812	-	379	15675795	spy2009	-	2,3,6,12	hypothetical protein
14	1676909	1680454	-	1181	15675796	spy2010	*scpA*	All	**C5A peptidase precursor**
15	1680888	1682093	+	401	15675797	spy2013	-	2,9,10	IS1562 transposase
16	1682672	1683598	-	308	15675798	spy2016	*sic*	2,9,10	**inhibitor of complement-mediated lysis**
17	1683787	1685241	-	484	15675799	spy2018	*emm1*	All	**M protein type 1**
18	1685426	1687015	-	529	15675800	spy2019	*mga*	All	**M protein trans-acting positive regulator**

Start -> 1663706 **Region defined by the TA skew analysis**

Start -> 1665608 **Missing region in the M-protein-neg strain T11**

19	1687691	1687942	-	83	15675801	spy2023	-	All	**hypothetical protein (mga-associated)**		
20	1688021	1689646	-	541	15675802	spy2025	*isp*	All	**immunogenic secreted protein precursor**		
21	1689748	1691136	-	462	15675803	spy2026	-	All	putative histidine kinase		
22	1691133	1691786	-	217	15675804	spy2027	-	All	putative two-component response regulator		
23	1691880	1693097	-	405	15675805	spy2029	-	All	putative ABC transporter (ATP-binding protein)		
24	1693110	1693784	-	224	15675806	spy2031	-	All	putative ABC transporter (ATP-binding protein)		
25	1693771	1695039	-	422	15675807	spy2032	-	All	putative ATP-binding cassette transporter-like protein		
26	1695462	1695866	-	134	15675808	spy2033	-	All	hypothetical protein		
27	1695893	1696189	-	98	15675809	spy2034	-	All	conversed hypothetical protein		
28	1696945	1697874	-	309	15675810	spy2037	*prsA*	All	peptidylprolyl isomerase		
29	1698661	1699857	-	398	15675811	spy2039	*speB*	All	**pyrogenic exotoxin B**		
30	1700129	1700299	-	56	15675812	spy2040	-	3,4,5,6,7,8,9,10,11,12	hypothetical protein		
31	1700383	1700514	+	43	15675813	spy2041	-	3,5,6,7,8,9,10,11,12	hypothetical protein		
32	1700798	1701640	+	280	15675814	spy2042	*ropB*	All	putative transcription regulator		
33	1701881	1702696	-	271	15675815	spy2043	*mf*	All	**mitogenic factor**		
34	1703060	1703569	+	169	15675816	spy2045	-	All	protein low temperature requirement C		
35	1703651	1704739	-	362	15675817	spy2047	*gldA*	All	glycerol dehydrogenase		**End -> 1708520**
36	1704796	1705464	-	222	15675818	spy2048	*mipB*	All	fructose-6-phosphate aldolase	**End -> 1710243**	
37	1705477	1707894	-	805	15675819	spy2049	*pflD*	All	putative pyruvate formate-lyase 2		
38	1708104	1709408	-	434	15675820	spy2050	-	All	putative PTS system, enzyme IIC component		
39	1709418	1709726	-	102	15675821	spy2051	-	All	putative PTS system, enzyme IIB		
40	1709754	1710074	-	106	15675822	spy2052	-	All	putative PTS system, enzyme III		

*Forty coding genes are present within the boudaries revealed by TA skew analyses. Ten out of the 40 (25%) genes are coding for known virulence factors and represents 24% of all known virulence genes in M1 SF370 (bold). Boundaries of the natural isolate of M-protein-negative group A streptococcus T11 are shown and closely correspond to those obtained with the TA skew. Information of proteins shared by SF370 and the other eleven sequences is given by numbers corresponding to the index of Table 1. Proteins common to all genomes are labeled as “All”.
